# *Bombyx mori* P-element Somatic Inhibitor (*BmPSI*) Is a Key Auxiliary Factor for Silkworm Male Sex Determination

**DOI:** 10.1371/journal.pgen.1006576

**Published:** 2017-01-19

**Authors:** Jun Xu, Shuqing Chen, Baosheng Zeng, Anthony A. James, Anjiang Tan, Yongping Huang

**Affiliations:** 1 Key Laboratory of Insect Developmental and Evolutionary Biology, Institute of Plant Physiology and Ecology, Shanghai Institutes for Biological Sciences, Chinese Academy of Sciences, Shanghai, China; 2 University of Chinese Academy of Sciences, Beijing, China; 3 Departments of Microbiology & Molecular Genetics and Molecular Biology & Biochemistry, University of California, Irvine, California, United States of America; The University of North Carolina at Chapel Hill, UNITED STATES

## Abstract

Manipulation of sex determination pathways in insects provides the basis for a wide spectrum of strategies to benefit agriculture and public health. Furthermore, insects display a remarkable diversity in the genetic pathways that lead to sex differentiation. The silkworm, *Bombyx mori*, has been cultivated by humans as a beneficial insect for over two millennia, and more recently as a model system for studying lepidopteran genetics and development. Previous studies have identified the *B*. *mori* Fem piRNA as the primary female determining factor and *BmMasc* as its downstream target, while the genetic scenario for male sex determination was still unclear. In the current study, we exploite the transgenic CRISPR/Cas9 system to generate a comprehensive set of knockout mutations in genes *BmSxl*, *Bmtra2*, *BmImp*, *BmImp*^*M*^, *BmPSI* and *BmMasc*, to investigate their roles in silkworm sex determination. Absence of *Bmtra2* results in the complete depletion of *Bmdsx* transcripts, which is the conserved downstream factor in the sex determination pathway, and induces embryonic lethality. Loss of *BmImp* or *BmImp*^*M*^ function does not affect the sexual differentiation. Mutations in *BmPSI* and *BmMasc* genes affect the splicing of *Bmdsx* and the female reproductive apparatus appeared in the male external genital. Intriguingly, we identify that *BmPSI* regulates expression of *BmMasc*, *BmImp*^*M*^ and *Bmdsx*, supporting the conclusion that it acts as a key auxiliary factor in silkworm male sex determination.

## Author Summary

The sex determination system extremely diverse among organisms including insects in which even each order occupy a different manner of sex determination. The silkworm, *Bombyx mori*, is a lepidopteran model insect with economic importance. The mechanism of the silkworm sex determination has been in mystery for a long time until a Fem piRNA was identified as the primary female sex determinator recently. However, genetic and phenotypic proofs are urgently needed to fully exploit the mechanism, especially of the male sex determination. In the current study, we provided comprehensively genetic evidences by generating CRISPR/Cas9-mediated knockout mutations for those genes *BmSxl*, *Bmtra2*, *BmImp*, *BmImp*^*M*^, *BmPSI* and *BmMasc*, which were considered to be involved in insect sex determination. The results showed that mutations of *BmSxl*, *BmImp* and *BmImp*^*M*^ had no physiological and morphological effects on the sexual development while *Bmtra2* depletion caused *Bmdsx* splicing disappeared and induced embryonic lethality. Importantly, the *BmPSI* regulates expression of *BmMasc*, *BmImp*^*M*^ and *Bmdsx*, supporting the conclusion that it acts as a key auxiliary factor to regulate the male sex determination in the silkworm.

## Introduction

Genetic systems for sex determination in insects show high diversity in different species. Sex determination in the fruit fly, *Drosophila melanogaster*, is controlled hierarchically by X:A > *Sxl* > *tra*/*tra2* > *dsx* and *fru* [1, 2]. The X:A ratio of 1 promotes transcription of *Sex-lethal* (*Sxl*) and results in feminization, while 0.5 to *Sxl* suppression and male differentiation [3–10]. *Sxl* proteins control the splicing of female *transformer* (*tra*) mRNAs that give rise to functional proteins, while no functional *Sxl* proteins exist in the male [11,12]. The search of homolog genes in the sex determination of *D*. *melanogaster* has been found a conserved relationship among *dsx*/*tra* across dipterans [13, 14] In another *Diptera*, *Musca domestica*, female employs a *F*^*D*^ allele which is encoded by the *tra* gene [15]. The medfly, *Ceratitis capitata*, has an as yet unidentified dominant male-determining factor on the Y chromosome [16]. The sex determination factors (F or M) in these two insects control the downstream gene *doublesex* (*dsx*) to generate sex-specific splicing isoforms. In contrast to *Drosophila*, *Cctra* and *Mdtra* seem to initiate an autoregulatory mechanism in XX embryos that provides continuous *tra* female-specific function and acts as a cellular memory maintaining the female pathway [17–19]. Many other insect species also exploit *tra* as the sex determination factor. For example, the honeybee, *Apis mellifera*, uses the *complementary sex determiner gene* (*csd*) to regulate feminization, which activates the *feminizer* gene (*fem*) by directing splicing to form the female functional Fem protein [20]. This *fem* gene is considered an orthologue of *Cctra* gene [21]. The *tra* gene in the red flour beetle, *Tribolium castaneum*, controls female sex determination by regulating *dsx* sex-specific splicing [22]. Also, *Lucilia cuprina* and *Nasonia vitripennis*, are reported to use *tra* as a female-determining signal [23, 24]. Recently, Hall et al. identified *Nix* a distant homolog of *transformer2* (*tra2*) from *Aedes aegypti* as the male-determining factor [25]. These data shows that the *dsx*/*tra* axis is conserved in many insect species and *tra* is the key gene around which variation in sex determining mechanisms has evolved in all insect species with the exception of *Aedes* and Lepidopteran insects.

Species of lepidoptera exhibit markedly different sex determination pathways from those seen in the flies, bees and beetles [26]. In the silkworm *Bombyx mori*, females possess a female-determining W chromosome have heteromorphic sex chromosomes (ZW) and males are homomorphic (ZZ) [27]. No *tra* ortholog has been identified in this order, possibly as a result of highly divergent sequence [28]. Bioinformatic analyses fail to identify a *tra* ortholog in *B*. *mori* and no *dsx*RE (*dsx* cis-regulatory element) binding sites are found in the target gene ortholog, *Bmdsx*, resulting in the default mode of female-specific splicing of the latter [29, 30]. *BmPSI* (P-element somatic inhibitor) and *BmHrp28* (hnRNPA/B-like 28) were reported to regulate *Bmdsx* splicing through binding CE1 sequences of the female-specific exon 4 of *Bmdsx* pre-mRNA [30, 31]. Another potential regulator, *BmImp* (IGF-II mRNA binding protein), enhances the male-specific splicing of *Bmdsx* pre-mRNA by increasing RNA binding activity of *BmPSI* [32]. The Z-linked *BmImp* binds to the A-rich sequences in its own pre-mRNA to induce the male-specific splicing of its pre-mRNA, and this splicing pattern is maintained by an autoregulatory mechanism, being *BmImp*^*M*^ (the male-specific splicing form of *BmImp*) able to bind its corresponding pre-mRNA [33]. Since *BmPSI* or *BmImp* products do not exhibit any sequence similarities to known Ser/Arg (SR) proteins, such as Tra and Tra2, the regulatory mechanisms of sex-specific alternative splicing of *Bmdsx* that of exon skipping is distinct from that of *Dmdsx* that of 3’ alternative splice[34]. Recently, the product of the W chromosome-derived *B*. *mori* sex determination factor *fem* (female-enriched PIWI-interacting RNA) was identified to target the downstream gene *BmMasc* for controlling *Bmdsx* sex-specific splicing [35]. This remarkable finding reveals that *fem* is the primary female sex determinator in the silkworm. However, the genetic relationship among these genes in *B*. *mori* sex determination is still in mystery.

We use here a binary transgenic CRISPR/Cas9 system to generate somatic mutations in sex determination pathway genes in *B*. *mori*. Three genes, *BmMasc*, *BmPSI* and *BmImp*, are involved in sex regulation only in lepidopteran insects, and two, *Bmtra2* and *BmSxl*, are structural orthologs of the key sex regulation factors in *Drosophila*. We focus on the sexually dimorphic traits of reproductive structures and sex-specific alternative splicing forms of *Bmdsx*, the bottom gene of the sex determination. The results show that *BmPSI* and *BmMasc* affect *Bmdsx* splicing and the male reproductive tissues, supporting the conclusion that they have roles in sex determination. Disruption of *BmImp* or *Bmtra2* causes severe developmental defects in both sexes, supporting their critical roles other than sex determination. Furthermore, loss-of-function mutations of *BmPSI* altered transcriptional or post-transcriptional (splicing) of *BmMasc*, *BmImp* and *Bmdsx* in males. These data strongly support the conclusion that *BmPSI* plays a key auxiliary in male sex determination in *B*. *mori*.

## Results

### CRISPR/Cas9-mediated mutagenesis of sex determination genes

**Fig 1:**
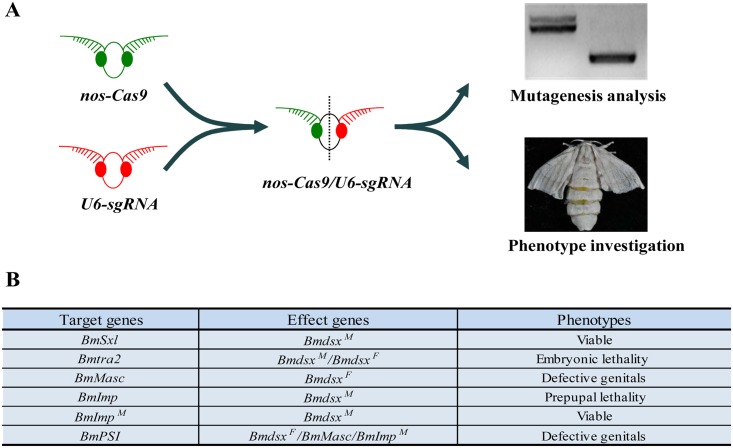
Loss-of-function analysis of *B*.*mori* sex determination genes. (A) Schematic representation of functional analysis using the transgenic CRISPR/Cas9 system. *nos-Cas9* (green) transgenic moths were crossed with *U6-sgRNA* (red) transgenic moths and the F_0_ heterozygotes were analyzed as for genotypic and phenotypic effects. (B) Genes affected and major phenotypic effects observed in mutants.

We established a binary transgenic CRISPR/Cas9 system to make somatic mutagenesis targeting selected genes. This system contains two separated lines, one is to express Cas9 protein under the control of a *B*. *mori nanos* promoter (*nos-Cas9*) and another is to express sequence-specific sgRNAs under the control of a *B*. *mori* U6 promoter (*U6-sgRNA*). Two independent lines of *nos-Cas9* and from three to 29 independent lines of each *U6-sgRNA* construct were obtained following *piggyBac*-mediated transgenesis (S1 Table). Genomic mutagenesis in F_0_ animals was confirmed by genomic PCR, and subsequent physiological phenotypes were investigated (Fig 1). The results confirm that the transgenic CRISPR/Cas9 system works effectively (S1–S5 Figs).

### *BmPSI* regulates transcription of *BmMasc* and *BmImp*^*M*^

**Fig 2:**
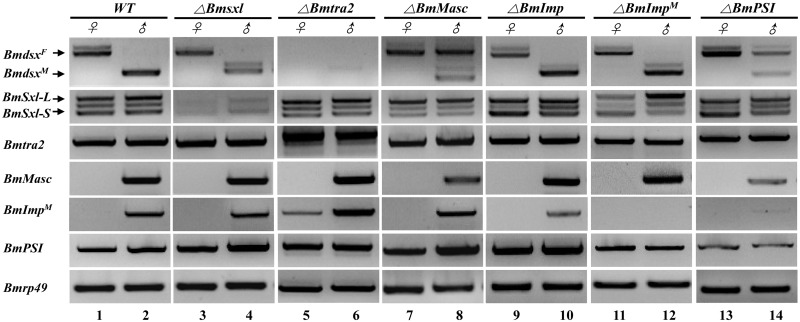
PCR-based gene amplification analyses for targeted genes in wild type (WT) and mutant animals. Six genes, *BmSxl*, *Bmtra2*, *BmMasc*, *BmImp*, *BmImp*^*M*^, and *BmPSI*, were mutated and corresponding expression files were listed. Expression of *Bmdsx* was investigated as a general indicator. *Bmdsx*^*F*^ and *Bmdsx*^*M*^ represent the female- and male-specific splicing isoforms of *Bmdsx*, respectively. *BmSxl-L* and *BmSxl-S* represent two different isoforms of the *BmSxl* gene. The lower panel shows amplification of the *rp49* transcript, which serves as an internal control for RNA extraction and RT-PCR.

*Bmdsx* is the conserved downstream component of the silkworm sex determination pathway and mutations in *BmPSI* and *BmMasc* had an effect on its splicing. Mutations in *BmSxl*, *BmImp* and *BmImp*^*M*^ resulted in the appearance of a larger *Bmdsx*^*M*^ (the male-specific splicing form of *Bmdsx*) mRNA isoform while the profile of *Bmdsx*^*F*^ (the female-specific splicing form of *Bmdsx*) appeared unaltered (Fig 2, lanes 4, 10 and 12). Sequence analysis of the larger *Bmdsx*^*M*^ isoform revealed an 81base-pairs (bp) length fragment insertion, creating a novel splice variant (S6 Fig). All *Bmdsx* isoforms were absent in both sexes in individuals carrying mutations in *Bmtra2* (Fig 2, lanes 5 and 6). Mutations in *BmMasc* and *BmPSI* in males resulted in a decreased accumulation of *Bmdsx*^*M*^ and the appearance of *Bmdsx*^*F*^ (Fig 2, lanes 8 and 14), which was consistent with previous report when *BmMasc* expression was disrupted by using RNAi [35].

**Fig 3:**
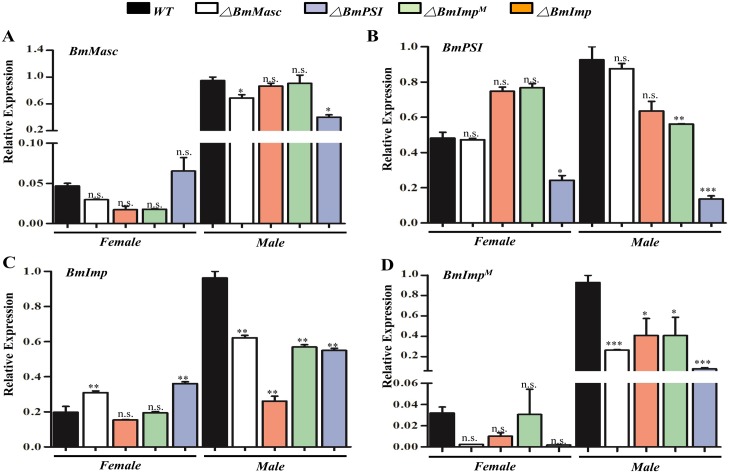
Q-RT-PCR analysis for investigation of *BmMasc*, *BmImp*^*M*^, *BmImp* and *BmPSI* transcript abundance in corresponding mutants. (A-D) Relative mRNA expression levels of *BmMasc*, *BmPSI*, *BmImp* and *BmImp*^*M*^ in mutant males and females, respectively. Three individual biological replicates were performed in q-RT-PCR. Error bar: SD; *, ** and *** represented significant differences at the 0.05, 0.01, 0.001 level (t-test) compared with the control.

RT-PCR-based analysis revealed that mutations in *BmSxl*, *BmMasc*, *BmImp* and *BmImp*^*M*^ had no or minor effect on other genes at transcriptional or post-transcriptional (splicing) levels except *Bmdsx*. The male-specific *BmImp* (*BmImp*^*M*^) isoform appeared in female *Bmtra2* mutants (Fig 2, lanes 5 and 6). This supports the conclusion that *Bmtra2* is not only involved in regulating *Bmdsx*, but also has a role in splicing regulation of *BmImp*^*M*^. Interestingly, the abundance of *BmMasc* and *BmImp*^*M*^ transcripts decreased in *BmPSI* mutant males (Fig 2, lane 14). Q-RT-PCR analysis showed that *BmImp*^*M*^ and *BmMasc* mRNA levels decreased by 92% and 60%, respectively, in *BmPSI* mutant males (Fig 3). These results support the conclusion that *BmPSI* regulates *BmMasc* and *BmImp*^*M*^ at the splicing level. In contrast to the results in males, with the exception of *Bmtra2*, no effects on transcript profiles were seen in any of the mutant females.

### *BmPSI* and *BmMasc* control male sexual differentiation

**Fig 4:**
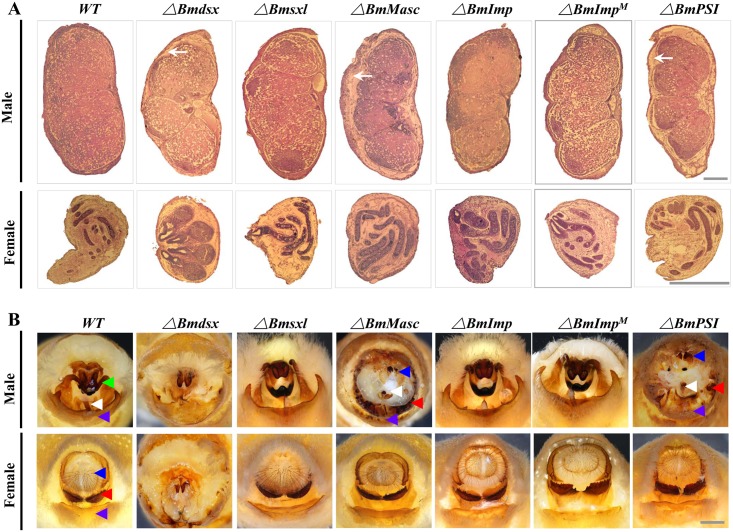
Morphological changes in sexually-dimorphic reproductive system structures in wild-type (WT) and mutant silkworms. (A) Morphology of internal gonad structure in paraffin sections stained with hematoxylin and eosin. Gonads are dissected from wild-type and mutant males and females on the fourth day of the fifth-instar. White arrows show the separation of the testis envelope from the testicular lobe. Scale bars: 250μm. (B) Gross morphology of external genitalia of control and mutant adults. Key to arrows: red, ventral chitin plate; blue, genital papillae; white, penis; green, clasper; and purple, ventral plates. Scale bars: 1mm.

**Fig 5:**
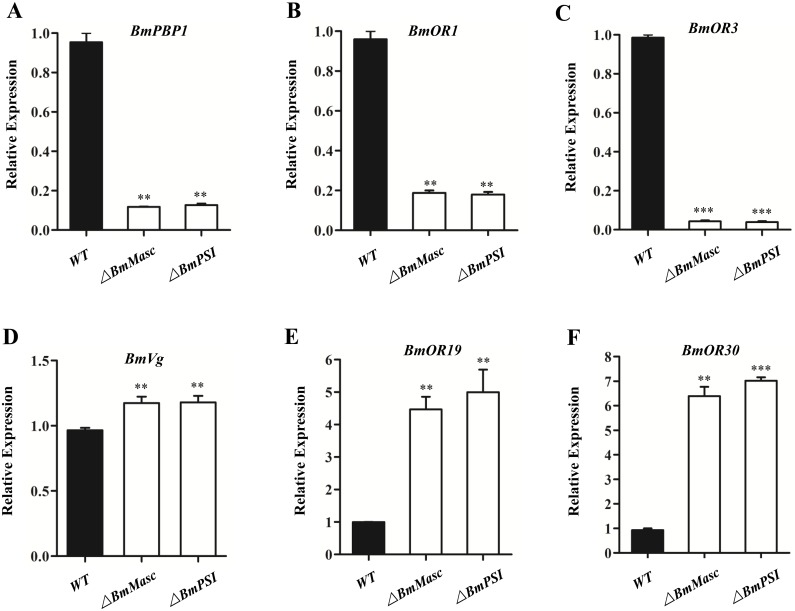
Q-RT-PCR analysis of the putative downstream genes of *Bmdsx* in the *BmMasc* and *BmPSI* mutants. (A-F) Relative mRNA expression levels of *BmPBP1*, *BmOR1*, *BmOR3*, *BmVg*, *BmOR19 and BmOR30* in mutant males. Three individual biological replicates were performed in q-RT-PCR. Error bar: SD; ** and *** represented significant differences at the 0.01, 0.001 level (t-test) compared with the control.

The morphology of the external and internal genitalia provides a direct index of sexual differentiation in *B*. *mori*. Mutations in *BmMasc* result in males with degenerative testes similar to that observed with *Bmdsx* mutants (Fig 4A, lanes 2 and 4). The external genitalia of males exhibit characteristics of the copulatory organs of both males and females including the female-specific ventral chitin plate and genital papillae (Fig 4B, lanes 2 and 4). The testes and external genitalia of mutant *BmPSI* males are similar phenotypes of mutations in *Bmdsx*^*M*^ or *BmMasc* and result in male sterility (Fig 4A, lane 7; Fig 4B, lane 7). The external and internal genitalia appear normal in both males and females with mutations in *BmSxl*, *BmImp*, and *BmImp*^*M*^, (Fig 4A, lanes 3, 5, 6; Fig 4B, lanes 3, 5, 6). The putative *dsx* target male-specific expression genes in the male olfactory system, *pheromone binding protein 1* (*BmPBP1*), *olfactory receptors* (*BmORs*) *BmOR1*, *BmOR3*, were significantly down-regulated in the *BmMasc* and *BmPSI* male mutants (Fig 5A–5C). In contrast, the female-specific expression genes in the female oogenesis and olfactory system, *vitellogenin* (*BmVg*), *BmOR19*, *BmOR30*, were significantly up-regulated in the *BmMasc* and *BmPSI* male mutants (Fig 5D–5F). These morphological results and corresponding gene expression profiles provide additional support for the conclusion that *BmPSI* and *BmMasc* control male sexual differentiation.

### *Bmtra2* and *BmImp* have pleiotropic effects in development

**Fig 6:**
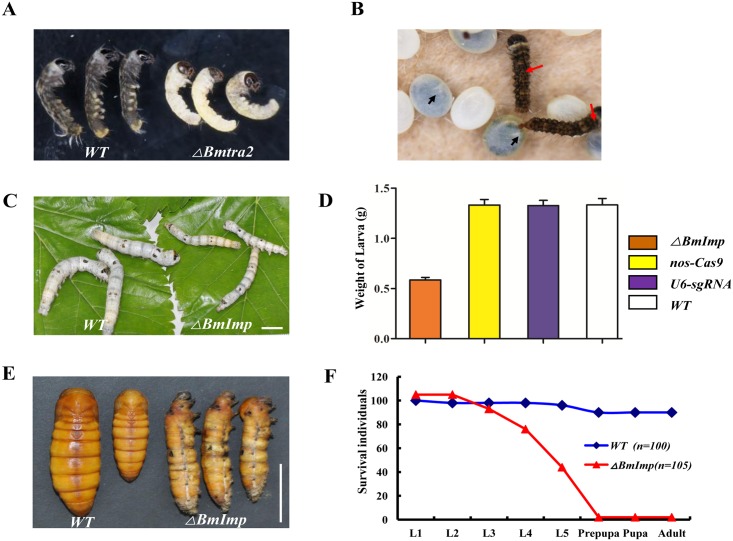
Pleiotropic effects of mutations in the *Bmtra2* and *BmImp* genes. (A and B) Embryonic lethality in progeny of the mating of *nos-Cas9* and *U6-Bmtra2-sgRNA*: F_0_ individuals complete embryogenesis (A) but fail to hatch (B). Embryos appear intact at eight days after oviposition and died on the twelfth day. Red arrows indicate control and black arrows indicate mutants. (C-F) Loss of *BmImp* function affects growth and metamorphic development. (C and D) Phenotypes of larvae resulted from crosses between *nos-Cas9* and *U6-BmImp-sgRNA* at the fifth day of the final larval instar. Smaller larvae are from mutant insects and larger larvae were from controls as showed in C. Scale bar: 1 cm. Data are from the crosses of *ΔBmImp* (*nos-Cas9*/*U6-sgRNA*), *nos-Cas9* (*nos-Cas9*/*-*), *U6-sgRNA* (*-*/*U6-sgRNA*) and WT (wild-type) as shown in D. E, Most larvae do not complete metamorphosis and die at the prepupal stage. Morphology of fifth instar larvae (*ΔBmImp*) compared to control. F, percent of wild-type (control, blue line) and mutant (*ΔBmImp*, red line) silkworms progressed through development. Key: L1-5, last day of the first, second, third and fourth larval instars, respectively. Data are from *ΔBmImp* (n = 105) and control (n = 100) animals.

Mutations in *Bmtra2* result in lethality at later embryonic stages (Fig 6A and 6B). This phenotype is similar to that reported in the honeybee in which down-regulation of *Amtra2* causes embryonic viability and affects the female-specific splicing of *fem* and *Amdsx* transcripts [36]. Also, in *T*. *castaneum*, only a few eggs could be produced by animals after the parental RNAi of *Tctra-2* and these eggs ultimately failed to hatch [37]. This is different from Dipteran species, in which *tra2* has no vital function in embryogenesis [38]. The similarity of these phenotypes supports the hypothesis that *Bmtra2* and its orthologs have an essential, ancestrally- and evolutionarily-conserved function in embryogenesis that is not related to sex determination that predates the divergence of the Lepidoptera, Hymenoptera and Coleoptera.

Two distinct types of mutations were induced in *BmImp*, the first one targets all splice variants (*BmImp*), and the second one is only in the male-specific splice variant (*BmImp*^*M*^) (S4 Fig). However, neither had an effect on the morphology of the genitalia despite the observed effect on *Bmdsx*^*F*^. While the growth indices of *BmImp*^*M*^ mutant silkworms were normal, the body size and weight of the *BmImp* mutants was smaller than wild-type animals (Fig 6C and 6D). They failed to molt at each larval instar and the majority died at the later larval and prepupal stages (Fig 6E and 6F).

## Discussion

**Fig 7:**
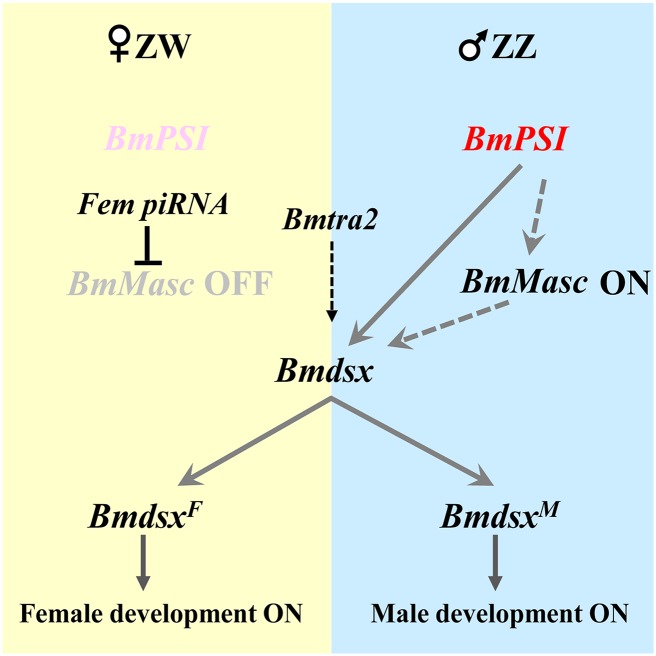
Proposed genetic cascade of sex determination in *B*. *mori*. The *Fem* piRNA derived from female W chromosome downregulates *Masc* expression (yellow panel) [27]. Absence of *Masc* products and inactive *BmPSI* result in splicing that produces the female-specific isoform of *Bmdsx* (*Bmdsx*^*F*^), which contain all of the exons of the gene. *Bmdsx*^*F*^ products induce female determination and differentiation of morphological sex characteristics. Thus, female development is the default sex determination pathway. *Masc* products are expressed normally in the male due to the lack of *Fem* and presence of active *BmPSI*, and this results in splicing of the male-specific isoform of *Bmdsx* (*Bmdsx*^*M*^, blue panel), which contain exons of 1, 2, 5 of the *Bmdsx* pre-mRNA. *Bmdsx*^*M*^ products promote male development. The *tra2* product also is involved in the regulation of *Bmdsx*.

We provide here genetic evidence for the proposed sex determination pathway in *B*. *mori* that emphasizes the key roles of the products of the *BmPS1* and *BmMasc* genes in male determination and differentiation (Fig 7). *B*. *mori* has the ZZ/ZW sex chromosome system in which the female is ZW (heterogametic) and the male is ZZ (homogametic). The *Fem* piRNA gene is located on the W chromosome and maintains feminization through downregulating *BmMasc* expression. Without the *BmMasc* protein in ZW embryos, the default (full-length) splicing isoform of *Bmdsx* (*Bmdsx*^*F*^) activates downstream gene expression and produces female-specific development. *BmPSI* is not involved in this pathway, since mutations in it have no observable effects on female differentiation. *BmPSI* protein in males interacts with the *Bmdsx* pre-mRNA and generates the male-specific *Bmdsx* splice variant (*Bmdsx*^*M*^) [30]. The *BmMasc* product might play the role of a recruitment or splicing factor to participate in this event. *BmMasc* is expressed normally in males due to lack of *Fem* piRNA, and thus may be controlled by *BmPSI*.

Significant differences are evident in the *B*. *mori* sex-determination system when compared to *D*. *melanogaster*. *Sxl* has a major and early role in sex determination in the fruit fly. Its ‘on/off’ status serves as the primary signal to trigger somatic sex differentiation by controlling its own splicing (autocatalytically) and of *tra* [38]. However, *Sxl* does not show sex-specific splicing and function in other dipterans [39]. Furthermore, the silkworm ortholog, *BmSxl*, has two splicing isoforms, neither of which is regulated in a sex-specific manner [40]. Depletion of *Bmsxl* induced a longer *Bmdsx*^*M*^ sex-specific splicing form appeared, just as seen in *BmImp* or *BmImp*^*M*^ disruption. Although the potential role of this splicing form was unclear, it did not bring any phenotypic consequence in male sexual dimorphism. The presence of a longer *Bmdsx*^*M*^ splicing form might because the spliceosome is complex machinery containing up to 300 proteins and loss of additional auxiliary factors having little roles [41, 42].

*Dmtra2* is a co-binding protein of sex determination key gene *Dmtra*, and acts on the *D*. *melanogaster* sex-determination cascade to regulate both somatic sexual differentiation and male fertility [43]. However, the ortholog encoding the *tra* appears to not exist in the silkworm, and therefore *Bmtra2* might have distinct roles. *Bmtra2* could regulate female-specific splicing of *Dmdsx* and RNAi knockdown of *Bmtra2* in the early embryo could cause abnormal testis formation in *B*. *mori* [44]. Our data support the conclusion that *Bmtra2* regulates *Bmdsx* in both males and females, and has sex-independently essential roles during embryogenesis although the mechanism of embryonic lethality induced by *Bmtra2* mutagenesis is not known. Another phenomena is *BmImp*^*M*^ appeared in the *Bmtra2* mutants, indicating that *Bmtra2* is involved in regulating the upstream transcriptional factors of *BmImp*^*M*^. It is similar with *Dmtra2* which regulates many downstream genes [45].

The *BmImp* gene was firstly identified in the silkworm as a co-binding protein with *BmPSI* [32]. *BmImp* produces two splice variants, one of which (*BmImp*^*M*^) is expressed in various tissues only in males and is proposed to be an essential regulator in the *B*. *mori* sex determination cascade [33]. *BmImp*^*M*^ could bind the *Bmdsx* pre-mRNA and knock-down of its product induced the female-specific *Bmdsx* splicing form in male cell lines and embryos [33, 46]. We cannot account for the differences in our results, but speculate that they could be a consequence of different methods between RNAi and Cas9-mediated gene silencing, or different materials between cell lines and the intact animal. However, our phenotypic results are consistent with those seen in *D*. *melanogaster* and the mouse. In the fruit fly, loss-of-function *Imp* mutations were zygotic lethal which through imprecise P excision, or mutants die later as pharate adults by two loss-of-function alleles, H44 and H149 [47, 48]. *Imp1* mutations in mice produced animals that were ~40% smaller than wild-type controls, and these exhibited high perinatal mortality [49]. Our experiments in which we mutate all isoforms of *BmImp* or only *BmImp*^*M*^ did not result in any effects on sexual organs or sex determination genes. We conclude that *BmImp* and *BmImp*^*M*^ are not essential for sex regulation but are needed for development.

*BmMasc* had been identified as a downstream target of *Fem*-piRNA which could induce *Bmdsx*^*F*^ in male embryos after siRNA-mediated knocking down [35]. *BmMasc* also controls dosage compensation in male embryos, and male embryos injected with *BmMasc* siRNA did not hatch normally [35]. Kiuchi *et al*. [35] used two siRNAs to knockdown *BmMasc* and reported detecting *Bmdsx*^*F*^ in male embryo and the down-regulation of *BmImp*^*M*^. They proposed that *BmImp*^*M*^ and *Bmdsx* are located downstream of *BmMasc* in the sex determination cascade. In contrast, our genetic analyses show no evidence of an effect of *BmMasc* on *BmImp*^*M*^. We propose that *BmMasc* controls male sexual differentiation by regulating *Bmdsx* but has no regulatory effect on *BmImp*^*M*^. It is still unclear how *BmMasc* regulates *Bmdsx*.

F or M factors have been identified as the *tra* or *tra2* gene in several insect species, and these factors directly regulated the *dsx* gene [25, 50]. Previous reports concluded that *BmPSI* could directly bind *Bmdsx* pre-mRNA and that the *BmImp*^*M*^ product increased *BmPSI* RNA binding activity *in vitro* [32]. Mutations of *DmPSI* in *D*. *melanogaster* strongly affect 43 splicing events and *DmImp* is one of the downstream genes targeted by it [51, 52]. We found that *BmImp* and *BmImp*^*M*^ had minor effects on *Bmdsx*^*F*^ splicing in our molecular genetic analyses. Furthermore, *BmPSI* did regulate expression of *Bmdsx*, *BmImp*^*M*^ and *BmMasc in vivo*. It is unknown how *BmPSI* regulates the splicing of *Bmdsx* and other potential splicing factors involved in this process. Nonetheless, these data support the conclusion that *BmPSI* is at least a key auxiliary factor in the silkworm male sexual differentiation gene cascade, and provide the basis for the hypothesis that the *BmPSI* gene has a major initial role in the sex determination cascade.

## Materials and Methods

### Silkworm strains

Silkworms of the same genetic background (Nistari, a multivoltine, nondiapausing strain) were used in all experiments. Wild-type (WT) and mutant larvae were reared on fresh mulberry leaves under standard conditions [53].

### Plasmid construction and germline transformation

A binary transgenic CRISPR/Cas9 system was established to target selected genes. The *piggyBac*-based plasmid, *pBac[IE1-EGFP-nos-Cas9]* (*nos-Cas9*), was constructed to express the Cas9 nuclease in germ-line cells under the control of the *B*. *mori nanos* (*nos*) promoter with the EGFP fluorescence marker gene under the control of the IE1 promoter. The plasmid pBac[IE1-DsRed2-U6-sgRNA] (*U6-sgRNA*) was constructed to express single guide RNAs (sgRNA) under the control of the silkworm U6 promoter and the DsRed fluorescence marker gene also under control of the IE1 promoter. The sgRNAs targeting sequences were designed by manually searching genomic regions that match the 5′-GG-N_18_-NGG-3′ rule [54]. sgRNA sequences were checked bioinformatically for potential off-target binding using CRISPRdirect (http://crispr.dbcls.jp/) by performing exhaustive searches against silkworm genomic sequences [55]. All sgRNA and oligonucleotide primer sequences for plasmid construction are listed in S2 Table. *U6-dsx-sgRNA* line from our previous report [56]. Each *nos-cas9* or *U6-sgRNA* plasmid mixed with a *piggyBac* helper plasmid [53] was microinjected separately into fertilized eggs at the preblastoderm stage. G_0_ adults were mated to WT moths, and resulting G_1_ progeny scored for the presence of the marker gene product using fluorescence microscopy (Nikon AZ100) (S1 Table).

### Genotyping and phenotypic analysis

Each U6-sgRNA transgenic line was mated individually with the *nos-Cas9* line to derive mutated F_0_ animals. Genomic DNA of mutated animals was extracted at the embryonic or larval stage using standard SDS lysis-phenol treatment after incubation with proteinase K, followed by RNase treatment and purification. The resulting individual DNA samples from mutant animals were separated by sex using gene amplification with primers specific to the W chromosome (S2 Table). For two sgRNA sites, mutation events were detected by amplification using gene-specific primers which set on the upstream or downstream of the each target (S2 Table). Amplified products were visualized by 2% agarose gel electrophoresis running 30 min at 100V. Amplicons were sub-cloned into the pJET-1.2 vector (Fermentas) and the positive clones of each we pick six were sequenced. For the one sgRNA, a restriction enzyme (*HpyAV*, New England Biolabs, Ipswich, MA, USA) cutting site adjacent to the AGG (PAM sequence) was selected to analyze the putative mutations by restriction enzyme digestion (RED) assay. The RED assay was carried out as previous report [57].

Phenotypic analysis focused light-microscope examination of the morphology of secondary sexual characteristics including internal and external genitalia. Photographs were taken with NRK-D90 (B) or DS-Ri1 (Nikon, Tokyo, Japan) digital cameras. Testes or ovaries were dissected from fourth-day, fifth-instar larvae and fixed overnight in Qurnah’s solution (anhydrous ethanol: acetic acid: chloroform = 6:1:3v/v/v). Tissues were dehydrated, cleared three times using anhydrous ethanol and xylene, respectively, and embedded in the paraffin overnight. Tissue sections (8 μm) were cut (Leica; RM2235) and stained using a mixture of hematoxylin and eosin solution. All pictures were taken under a microscope (Olympus BX51) using differential interference contrast with the appropriate filter.

### Qualitative and quantitative RT-PCR

Total RNA was extracted from silkworm at different stages using Trizol reagent (Invitrogen) and treated with RNase-free DNAse I (Ambion). cDNAs were synthesized using the Omniscript Reverse transcriptase kit (Qiagen) in a 20 μl reaction mixture containing 1 μg total RNA. RT-PCR reactions were carried out using KOD plus polymerase (Toyobo) with gene-specific primers (S2 Table). Amplification of the *B*. *mori* ribosomal protein 49 (*Bmrp49*) was used as a positive control.

Quantitative real-time RT-PCR (Q-RT-PCR) assays were performed using SYBR Green Realtime PCR Master Mix (Thermo Fisher Scientific) on an Eppendorf Real-time PCR System Mastercycler realplex (Eppendorf). Q-RT-PCR reactions were carried out with gene-specific primers (S2 Table). A 10-fold serial dilution of pooled cDNA was used as the template to make standard curves. Quantitative mRNA measurements were performed in three independent biological replicates and normalized to *Bmrp49* mRNA.

### Statistics

Experimental data were analyzed with the Student’s t-test. t-test: *, p < 0.05, **, p < 0.01,***, p < 0.001. At least three independent replicates were used for each treatment and the error bars show means ± S.E.M.

## Supporting Information

S1 Table Oligonucleotide primers used in this study. (XLSX)

S2 Table Features and numbers of transgenic strains used in this study. (XLSX)

S1 Fig A binary transgenic CRISPR/cas9 system induces mutations at the *BmSxl* locus in *B*. *mori*. (**A**) Schematic representation of the exon/intron boundaries of the *BmSxl* gene. Exons are shown as boxes. Untranslated regions are shown as black boxes and coding regions as open boxes. Thin lines represent the intron and numbers are the lengths in kilobase-pairs (kb). Target site locations are noted and PAM sequences are shown in red. (**B**) The binary transgenic CRISPR/Cas9 system in this study contains two lines, one contains the full Cas9 ORF driven by the *nanos* (*nos*) promoter, and another contains one U6 promoter-driven sgRNA. These two lines also have the reporter genes *EGFP* or *DsRed2*, respectively, under the control of the *IE1* promoter. (**C**) RED analysis in the WT and *Bmsxl* male and female mutants. (**D**) Various mutations were induced in F_0_ founder animals following crosses between *nos-Cas9* and *U6-BmSxl-sgRNA* strains. The targeting sequence is in blue and PAM sequence in red. The deletion size is indicated by the number of base pairs (bp).
(TIF)

S2 Fig Transgenic CRISPR/cas9 system induces mutations at the *Bmtra2* locus in *B*. *mori*. (**A**) Schematic representation of the exon/intron boundaries of the *Bmtra2* gene. (**B**) The binary transgenic CRISPR/Cas9 system targeting *Bmtra2*. (**C**) PCR analysis of *Bmtra2* male and female mutants. (**D**) Various mutations were induced in F_0_ founder animals following crosses between *nos-Cas9* and *U6- Bmtra2-sgRNA* strains.
(TIF)

S3 Fig Transgenic CRISPR/cas9 system induces mutations at the *BmMasc* locus in *B*. *mori*. (**A**) Schematic representation of the exon/intron boundaries of the *BmMasc* gene. (**B**) PCR analysis of *BmMasc* male and female mutants. (**C**) Various mutations were induced in F_0_ founder animals following crosses between *nos-Cas9* and *U6-BmMasc-sgRNA* strains.
(TIF)

S4 Fig Transgenic CRISPR/cas9 system induces mutations at two different loci of the *BmImp* in *B*. *mori*. (**A**) and (D) Schematic representation of the exon/intron boundaries of the *Imp* gene. (**B**) and (E) PCR analysis of *BmImp and BmImp*^*M*^ male and female mutants. (**C**) and (F) Various mutations were induced in F_0_ founder animals following crosses between *nos-Cas9* and *U6-BmImp-sgRNA* or *U6-BmImp*^*M*^*-sgRNA* strains, respectively.
(TIF)

S5 Fig Transgenic CRISPR/cas9 system induces mutations at the *BmPSI* locus in *B*. *mori*. (**A**) Schematic representation of the exon/intron boundaries of the *BmPSI* gene. (**B**) PCR analysis of *BmPSI* male and female mutants. (**C**) Various mutations were induced in F_0_ founder animals following crosses between *nos-Cas9* and *U6-BmPSI-sgRNA* strains.
(TIF)

S6 Fig Sequence of the extra amplicon of *Bmdsx*^*M*^. The longer transcript (*BmdsxM-L*) contains an 81bp fragment between the exon1 and exon2.
(TIF)

S7 Fig Transcription expression analysis of *Bmdsx* common region in *Bmtra2* mutants. RT-PCR amplification the exon1 of *Bmdsx* from different sexes of WT and mutant animals. Three male or female *Bmtra2* mutants were used (1–3). The lower panel shows amplification of the *rp49* transcript.
(TIF)

S8 Fig Relative mRNA expression of of *BmPSI* (A) and *BmMasc* (B) determined by using Q-RT-PCR at the different developmental stages of E20 (20 h at embryo stage after post-oviposition), E28, E32, E38, L1D3 (third day of first larvae instar), L3D1 (first day of third larvae instar), L4D1 (first day of fourth larvae instar), L5D3 (third day of fifth larvae instar), P (pupa stage), A (adult stage). The white bars indicate wild type females and the dot bars indicate wild type males. The results are expressed as the means±SD of three independent biological replicates.
(TIF)

S9 Fig Q-RT-PCR and RT-PCR analysis for investigation of *BmPSI*, *BmImp*^*M*^, *BmMasc*, and *Bmdsx* transcripts or splicing forms abundance in *BmPSI* mutant at different developmental stages. (**A-C**) Relative mRNA expression levels of *BmPSI*, *BmImp*^*M*^ and *BmMasc* in *BmPSI* mutant males and females at the embryonic (144 h after post-oviposition), larval (third day of fifth larvae instar) and pupal (the third day) stages, respectively. The white bars indicate wild type females and the dot bars indicate wild type males. The purple bars indicate mutant females and the dot bars with purple indicate mutant males. Three individual biological replicates were performed in q-RT-PCR. Error bar: SD; *, ** and *** represented significant differences at the 0.05, 0.01, 0.001 level (t-test) compared with the control. (**D**) PCR-based gene amplification analyses for *Bmdsx* splicing in *BmPSI* mutant animals. *Bmdsx*^*F*^ and *Bmdsx*^*M*^ represent the female- and male-specific splicing isoforms of *Bmdsx*, respectively. The lower panel shows amplification of the *rp49* transcript, which serves as an internal control for RNA extraction and RT-PCR.
(TIF)
