# Supplementary figures and images for: Bombyx mori P-element Somatic Inhibitor (BmPSI) Is a Key Auxiliary Factor for Silkworm Male Sex Determination
Source: PLoS Genet. 2017 Jan 19;13(1):e1006576. doi: 10.1371/journal.pgen.1006576 (PMC5289617; doi:10.1371/journal.pgen.1006576)

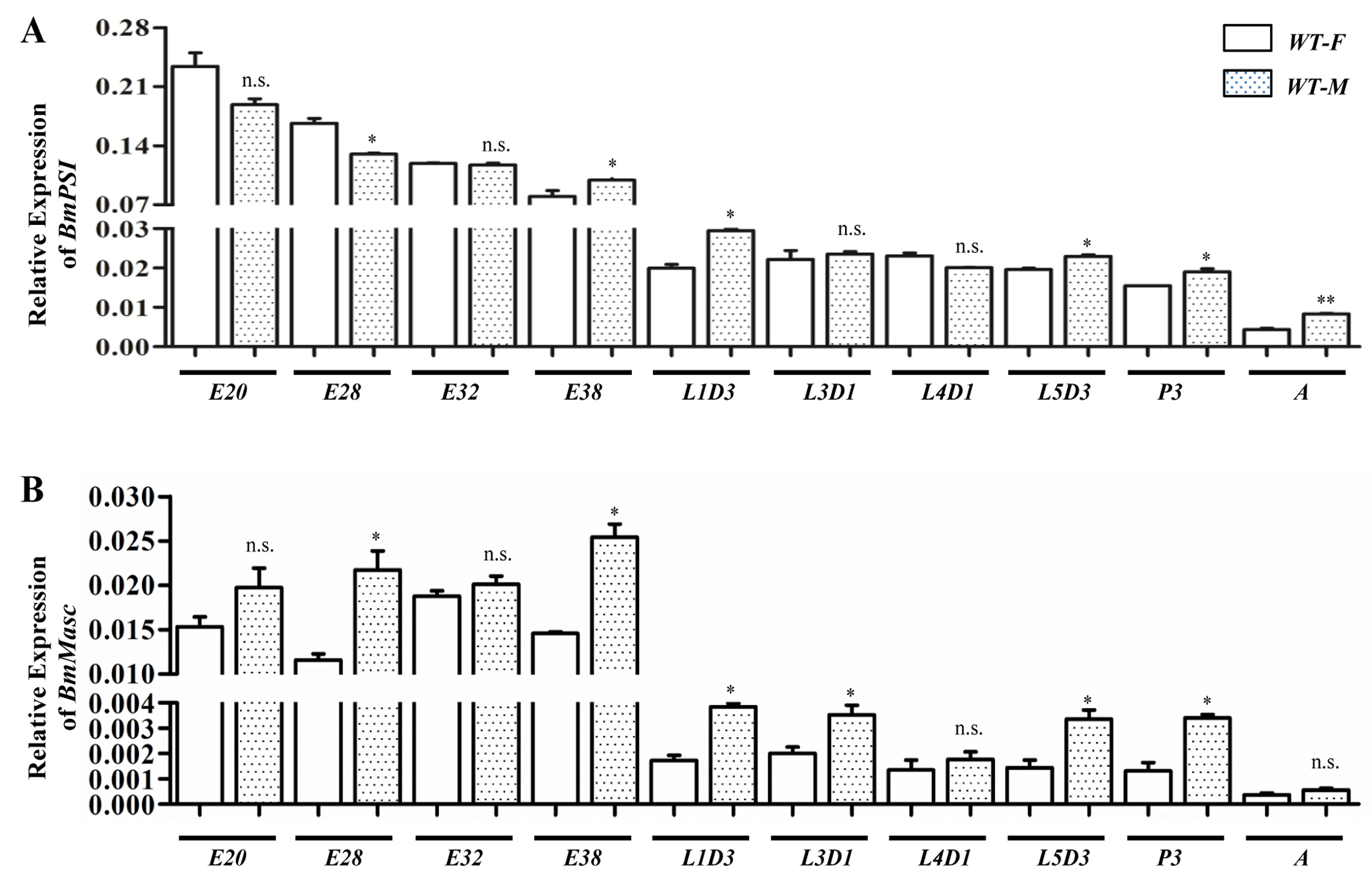

Supplement: S8 Fig — The white bars indicate wild type females and the dot bars indicate wild type males. The results are expressed as the means±SD of three independent biological replicates. (TIF) [file pgen.1006576.s010.tif]

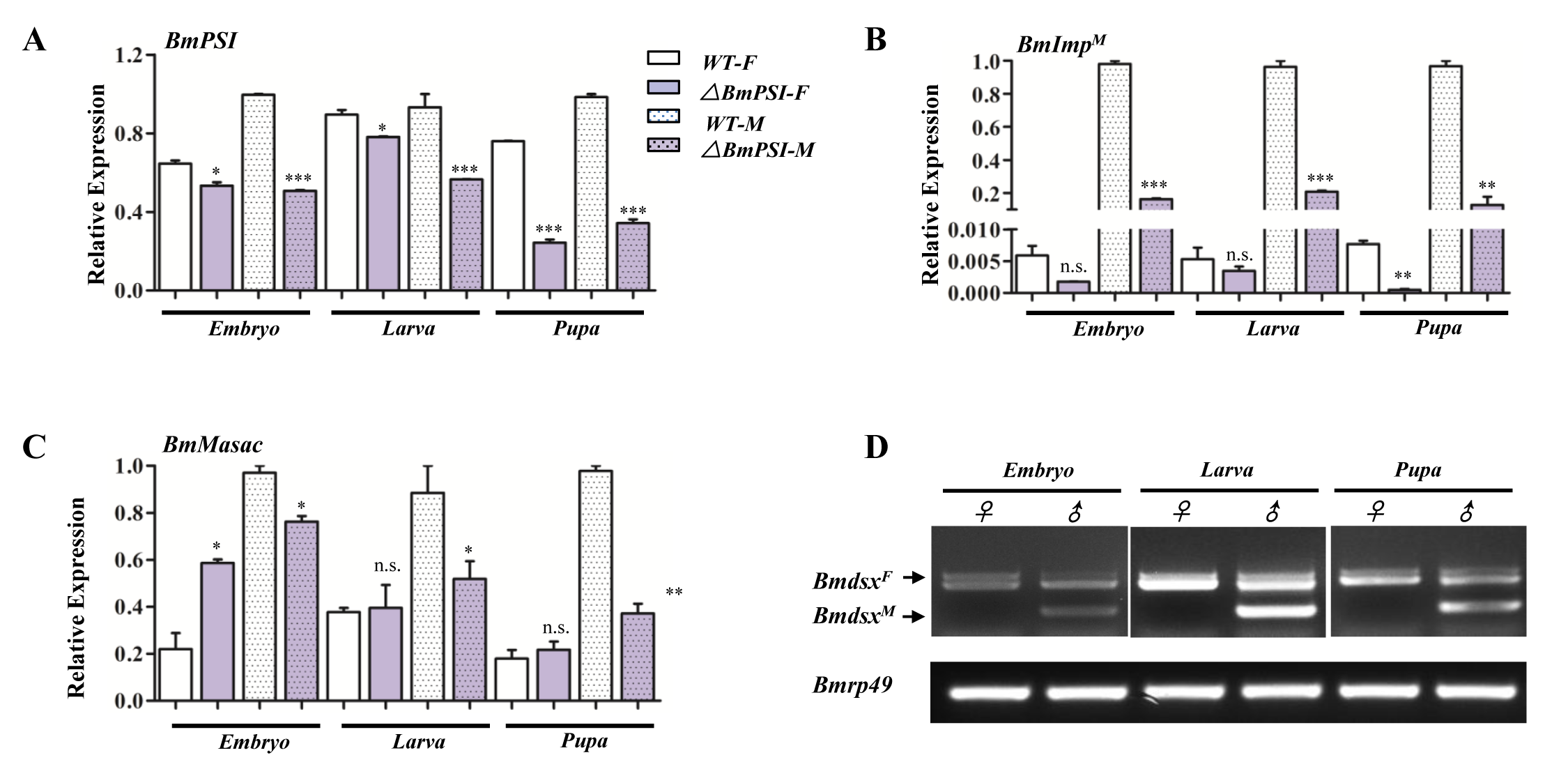

Supplement: S9 Fig — (A-C) Relative mRNA expression levels of BmPSI, BmImpM and BmMasc in BmPSI mutant males and females at the embryonic (144 h after post-oviposition), larval (third day of fifth larvae instar) and pupal (the third day) stages, respectively. The white bars indicate wild type females and the dot bars indicate wild type males. The purple bars indicate mutant females and the dot bars with purple indicate mutant males. Three individual biological replicates were performed in q-RT-PCR. Error bar: SD; *, ** and *** represented significant differences at the 0.05, 0.01, 0.001 level (t-test) compared with the control. (D) PCR-based gene amplification analyses for Bmdsx splicing in BmPSI mutant animals. BmdsxF and BmdsxM represent the female- and male-specific splicing isoforms of Bmdsx, respectively. The lower panel shows amplification of the rp49 transcript, which serves as an internal control for RNA extraction and RT-PCR. (TIF) [file pgen.1006576.s011.tif]
